# Feasibility Study of *Ex Ovo* Chick Chorioallantoic Artery Model for Investigating Pulsatile Variation of Arterial Geometry

**DOI:** 10.1371/journal.pone.0145969

**Published:** 2015-12-30

**Authors:** Kweon-Ho Nam, Juho Kim, Gicheol Ra, Chong Hyun Lee, Dong-Guk Paeng

**Affiliations:** 1 Interdisciplinary Postgraduate Program in Biomedical Engineering, Jeju National University, Jeju, South Korea; 2 Department of Ocean System Engineering, Jeju National University, Jeju, South Korea; University of Bari Medical School, ITALY

## Abstract

Despite considerable research efforts on the relationship between arterial geometry and cardiovascular pathology, information is lacking on the pulsatile geometrical variation caused by arterial distensibility and cardiomotility because of the lack of suitable *in vivo* experimental models and the methodological difficulties in examining the arterial dynamics. We aimed to investigate the feasibility of using a chick embryo system as an experimental model for basic research on the pulsatile variation of arterial geometry. Optical microscope video images of various arterial shapes in chick chorioallantoic circulation were recorded from different locations and different embryo samples. The high optical transparency of the chorioallantoic membrane (CAM) allowed clear observation of tiny vessels and their movements. Systolic and diastolic changes in arterial geometry were visualized by detecting the wall boundaries from binary images. Several to hundreds of microns of wall displacement variations were recognized during a pulsatile cycle. The spatial maps of the wall motion harmonics and magnitude ratio of harmonic components were obtained by analyzing the temporal brightness variation at each pixel in sequential grayscale images using spectral analysis techniques. The local variations in the spectral characteristics of the arterial wall motion were reflected well in the analysis results. In addition, mapping the phase angle of the fundamental frequency identified the regional variations in the wall motion directivity and phase shift. Regional variations in wall motion phase angle and fundamental-to-second harmonic ratio were remarkable near the bifurcation area. In summary, wall motion in various arterial geometry including straight, curved and bifurcated shapes was well observed in the CAM artery model, and their local and cyclic variations could be characterized by Fourier and wavelet transforms of the acquired video images. The CAM artery model with the spectral analysis method is a useful *in vivo* experimental model for studying pulsatile variation in arterial geometry.

## Introduction

Arterial geometry is among the main determinants of local hemodynamic forces, which are closely related to cardiovascular pathophysiology [1]. The information on the arterial geometric features is critical in developing prediction and treatment strategies for atherosclerosis; the estimation of focal regions with low wall shear stress (WSS), which facilitates the initiation and progression of atherosclerotic plaque, becomes possible with such information [2]. Many previous studies have investigated the hemodynamic-geometric etiology of atherosclerosis in the carotid [3–5] and coronary arteries [6–8] because such atherosclerosis events cause fatal cardiovascular diseases such as ischemic stroke and myocardial infarction. Zarins et al. [3] reported that the atherosclerotic plaques in the carotid artery preferentially develop at the posterior wall of the internal carotid artery because of the formation of flow recirculation zone associated with low WSS and high oscillatory shear index [9]. Based on this finding, numerous studies have been conducted to clarify the hemodynamic-geometric causes of the inter- and intra-individual differences in carotid atherosclerosis, which is currently known to be associated with geometric parameters of the carotid bifurcation, such as the cross-sectional area or diameter ratio of the internal to common carotid arteries [4, 10].

Computational fluid dynamics (CFD) models with rigid wall assumption have been widely used to estimate the spatio-temporal variations in arterial hemodynamic forces. The CFD simulation allows the 3D visualization of the detailed velocity field, from which WSS distribution 3D map could be obtained. Most CFD studies on arterial WSS have been performed under rigid wall conditions, which neglect arterial wall movements; however, some studies determined the effect of the arterial wall compliance on the blood flow structure and WSS [11–14]. Results of these studies indicate that arterial wall compliance does not significantly affect the global structure of the blood flow pattern, but significantly reduces the WSS magnitude. These results suggest that compliant wall modeling, known as fluid-structure interaction (FSI) analysis, closely reflects physiological conditions; hence, FSI analysis is necessary for *in vivo* estimation of the spatio-temporal distribution of local WSS [15]. However, FSI simulation has inherent limitations in analysis reliability because obtaining input information about the physical properties of arterial vessels and surrounding tissues is difficult and complicated by their inhomogeneity [16–18].

The relationship between arterial geometry variation and cardiovascular disorders continues to be an interesting research subject. However, experimental reports on the local variations of hemodynamics and WSS, which are measured by incorporating real wall motion of *in vivo* arteries, are limited both in clinical and basic studies because of the technological difficulties in imaging instantaneous changes in arterial geometry with high spatial and temporal resolutions. Ultrasound and magnetic resonance techniques can be used for this purpose, but such techniques have several drawbacks as follows. Ultrasound method has poor imaging quality because of low spatial resolution and various image artifacts [19]. Magnetic resonance imaging requires a long scanning time and is accompanied by loss of signal in turbulent regions [15], which reduce the measurement accuracy and reliability. The lack of basic and systematic studies on *in vivo* interaction between blood flow and artery wall is due to the difficulties in measuring pulsatile changes of arterial geometry. In addition, the feasibility of performing FSI simulation on them has not been sufficiently validated in an *in vivo* environment. To investigate the *in vivo* properties of the arterial geometry dynamics, a suitable *in vivo* experimental model that can easily visualize arterial motion and can provide various types of geometry, such as bifurcation and curvature, must be established.

Small animals, such as rats and mice, have been widely used as experimental models to investigate basic physiology and pathology. In investigating the characteristics of vascular structure and blood flow, small blood vessels in their mesenteries [20], and spinotrapezius [21] and cremaster muscles [22] were commonly used because these models are easy to prepare and the vessels can be well observed using optical microscope systems. However, even if the venous vessels in the models could be clearly observed, the arteries, especially large arteries, are usually deeply buried in the adipose tissues and muscles, thereby causing blurred images and low contrast between the vessel wall and surrounding tissues [23–24]. Therefore, small animal models are not suitable for basic research on arterial geometry variation.

In addition to the murine models, the chick embryo system has been used as an excellent model for basic study on circulatory system, such as microcirculation, angiogenesis, and cardiovascular development [25–28]. The chick embryo is a popular *in vivo* model in various research fields, such as biology, medicine and pharmacology, and has been extensively studied and well documented in numerous publications. The chick embryo system is receiving an increasing amount of attention because of its simplicity, rapid implementation, and low cost. In addition, using this model is relatively free from ethical and legal issues compared with murine models [29]. The chick embryo chorioallantoic membrane (CAM), an extraembryonic membrane that serves as the respiratory organ during embryo development, is particularly well suited for bright-field observation of various shapes of blood vessels because CAM provides a highly vascularized network within a transparent matrix [29–31]. The CAM model allows a longitudinal study because continuous visualization of blood vessels in the same embryo sample is possible. These advantages are highly beneficial for studying hemodynamics, blood vessel geometry, and vascular development. However, most previous studies on the CAM circulatory system were mainly based on the static geometry of veins and arteries, and the arterial geometry variation caused by blood pulsation was received less attention. We aimed to investigate the feasibility and usefulness of the CAM model to address the increasing need for an *in vivo* experimental model to study arterial wall motion. We obtained and analyzed video images of the chick CAM arteries with various shapes. The wall motion and geometrical dynamics of the arteries were characterized and visualized using image analysis techniques.

## Materials and Methods

### *Ex Ovo* Culture

All experimental procedures were approved by the Jeju National University Ethics Committee. Chicken eggs were purchased from commercial sources. The eggs were incubated in a digital incubator (RCOM PRO 50, Autoelex Co., South Korea) at 37.5°C and 70% humidity with turning at 90° angle once an hour. At Hamburger-Hamilton (HH) [32] stage 20 (3 days of incubation), the eggs were carefully cracked and the embryo samples were placed on a Petri dish. After covering with lids, the samples were further incubated until CAM developed rich blood vessels. More detailed descriptions of the culture method can be found elsewhere [33–34].

### Image Acquisition

**Fig 1:**
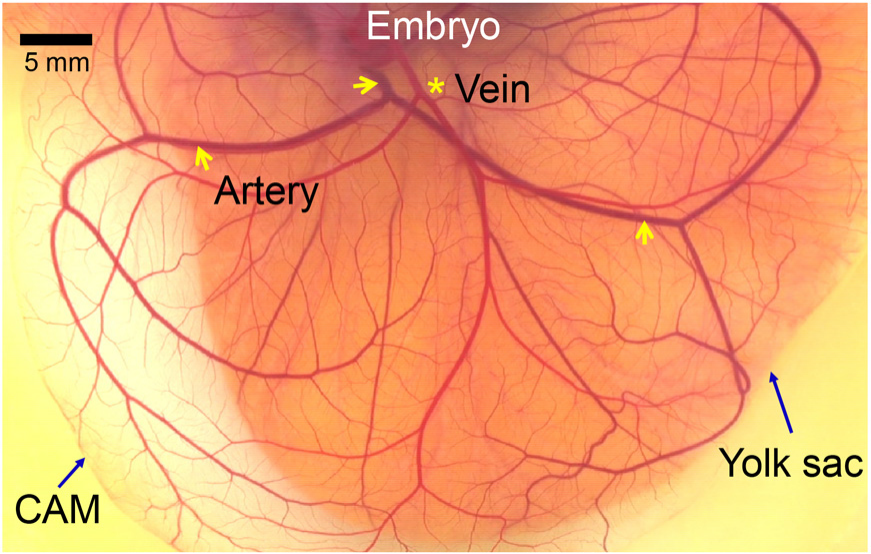
Photo of a chick embryo CAM at HH stage 36 (10 days of incubation). Transparent CAM with well developed vascular network over the yolk sac. An artery marked with arrows appears darker than vein marked with an asterisk.

The imaging system consists of a vertical microscope (Nikon ECLIPSE E200, Japan) with a halogen lamp and a digital video camera recorder (Sony HDR-XR520, Japan). An objective lens with a 4× magnification (numerical aperture = 0.10) was used. It is reported that the CAM is formed at 4 to 5 days of incubation, and increases in size and becomes more vascularized until day 11 when its growth rate remained minimal [29]. The video images of the CAM arteries were captured at a frame rate of 120 Hz for 3 seconds during well vascularized stages (HH stages 34–37 corresponding to 8–11 days of incubation). The pixel size of the microscopic images was approximately 2.4 μm/pixel. Imaging was performed at room temperature (25°C) in a thermostatic room. Fig 1 shows a typical image of the CAM vascular network at HH stage 36 (10 days of incubation). A main artery is bifurcated and further divided into numerous branches. The arteries can be easily identified from veins by motility and blood color of arteries. The microscopic artery images were captured from the downstream of the second level bifurcation because the CAM artery started from the bottom of the *ex ovo* culture system and was usually observed to float flat on the extraembryonic surface after the second-level bifurcation. Various images of arteries were obtained from more than 20 embryo samples, and the analysis results from seven representative video images were displayed.

## Vessel Boundary Detection

**Fig 2:**
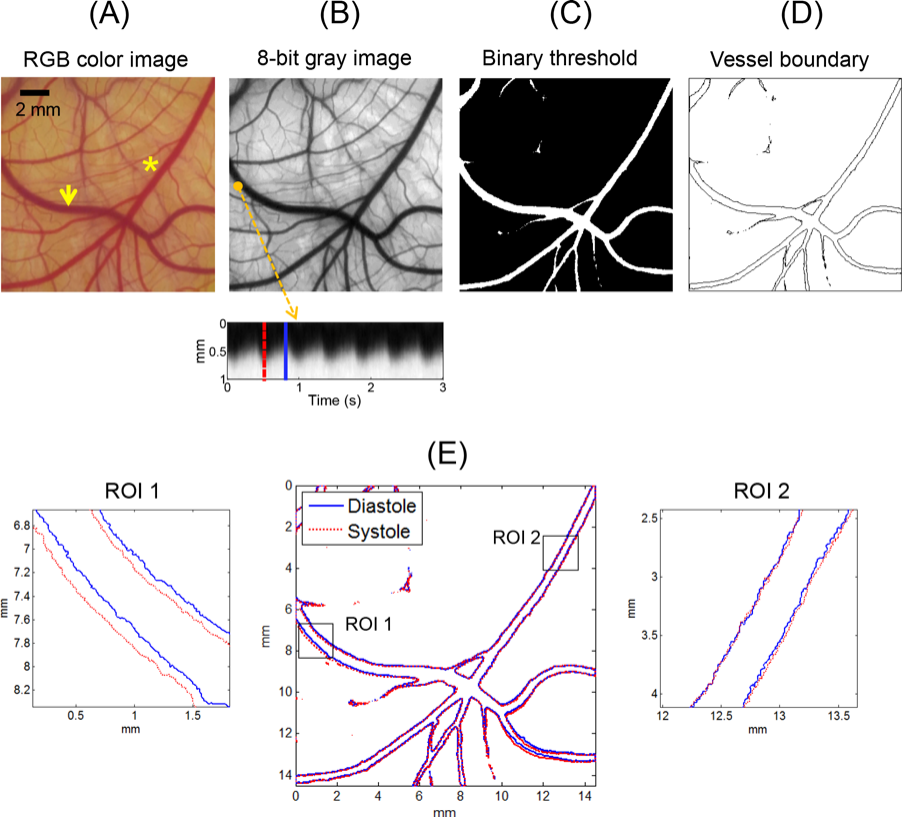
Schematic of vessel boundary detection. Vessel image was obtained from the CAM of an *ex ovo* sample at HH stage 37 (11 days). Arrow and asterisk in (A) indicate artery and vein, respectively. Bottom panel in (B) shows the time domain plot of image brightness variation at marked position (1 mm in vertical length). (E) Systolic and diastolic geometries were superimposed on a single image. Zoomed-in images of ROIs 1 and 2 are displayed in left and right panels, respectively.

Fig 2 shows the main steps of image analysis to detect vessel boundary. The original image in RGB color space was converted to an 8-bit gray image and binarized by thresholding the brightness values to classify areas of the background and the vessels (Fig 2A–2C). The threshold values were manually determined from the gray level information at the edge of the vessel of interest. Vessel boundaries were traced using the “bwboundaries” function in Matlab software (MathWorks, Natrick, MA, USA) that applies the Moore-Neighbor tracing algorithm modified by Jacob’s stopping criteria (Fig 2D) [35]. The systolic and diastolic phases were detected by time domain plotting of the arterial wall motion, as shown in Fig 2B (bottom panel). The vessel boundaries in the peak systolic and diastolic images corresponding to the selected frames presented as the dotted and solid lines in Fig 2B were detected and superimposed on a single frame (Fig 2E).

### Spectral Analysis of Pixel Brightness

The gray-level intensity of each pixel in an acquired video clip consisted of 360 consecutive images for 3 seconds was analyzed in frequency domain using a fast Fourier transform (FFT) algorithm. By FFT application, the relative magnitudes of the harmonic frequency components were calculated from the time-varying brightness profile of each pixel. The fundamental and second harmonic magnitudes, and their ratio were mapped to visualize the local variations in the harmonic components. The root mean square (RMS) amplitude was calculated from the detrended time domain data and mapped to estimate the relative variation in the local wall displacement during a pulsatile cycle. In addition, the phase angle of the fundamental frequency was calculated by taking the arctangent of the ratio between the imaginary and real parts of a complex number in the FFT output. The phase angle information was shown in a continuous phase map and a histogram. To simultaneously interpret the local wall motion in the time and frequency domains, we used the continuous wavelet transform (CWT), which allowed for higher resolution at high frequencies compared with the short time FFT method [36]. The Morlet wavelet function was used as the mother wavelet because this function is most popular in CWT and has been successfully used to analyze various physiological signals [37–39]. Analysis results were presented in a time-frequency spectrum called a scalogram. Detailed descriptions of the CWT analysis can be found elsewhere [38].

## Results

### The CAM Arteries

Fig 1 shows a typical CAM vascular network at HH stage 36 (10 days of incubation). The CAM artery was darker than the venous vessel because the venous blood is oxygenated in the extraembryonic circulation, in contrast to the blood in the internal circulatory system. The CAM arteries were easily distinguished from the venous vessels by the different blood color and vessel wall motion. The *ex ovo* samples at HH stages 34–37 (8–11 days of incubation) were suitable for use in investigating the arterial wall motion because the CAM vasculature was sufficiently developed during this period and showed various shapes and sizes of artery images.

### Systolic and Diastolic Geometry

**Fig 3:**
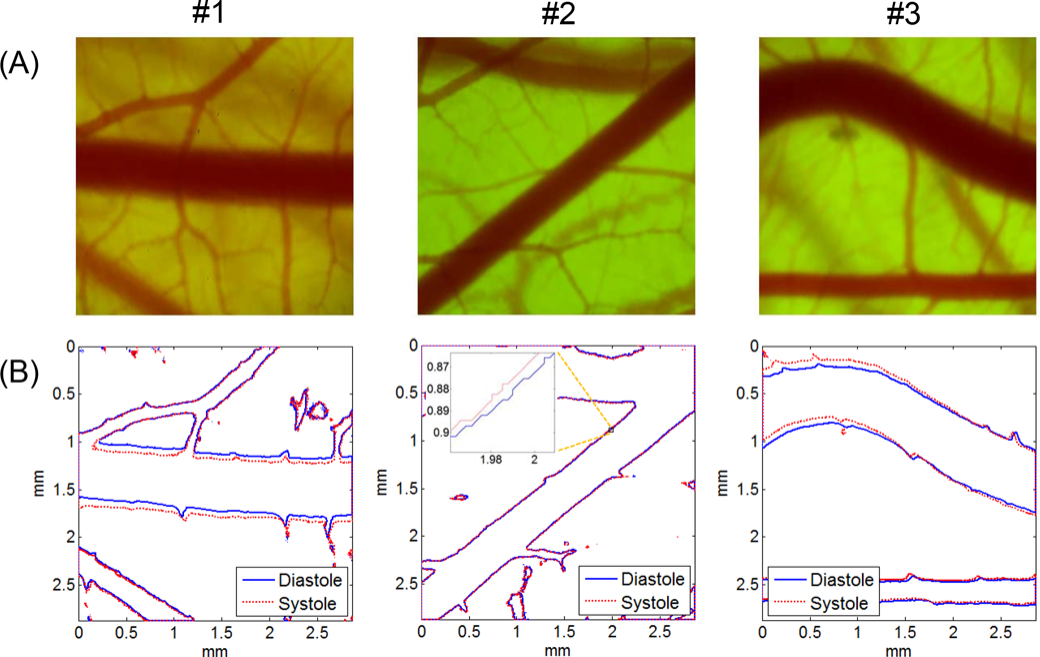
Microscopic CAM artery images and vessel boundaries at systole and diastole. (A) CAM artery images and (B) corresponding vessel boundaries detected at peak systole and late diastole. Artery images were obtained from three *ex ovo* samples (#1: HH 35; #2: HH 34; #3; HH 37). Microscope magnification was ×40. Image size was 1,200 pixels × 1,200 pixels.

Fig 2E presents a representative result of the boundary detection of the artery and vein captured in the same image. By superimposing the vessel boundaries at peak systole and late diastole on a single frame, the pattern of wall motion during a pulsatile cycle, such as the local variation of the wall movement direction and wall displacement amplitude, was well recognized visually. The zoomed-in images of the vessel boundaries in the artery (ROI 1) and vein (ROI 2) show that the artery had remarkable wall motion, but the wall displacement of the venous vessel was negligible. Fig 3 shows typical examples of microscopic CAM artery images obtained from three different *ex ovo* samples and the corresponding vessel boundaries observed at peak systole and late diastole. In case #1, the artery showed translational vessel movement in the up and down direction, whereas the arterial wall motion in case #2 was extremely weak and nearly unobservable. In the case of a curved artery (case #3), the analysis result showed large regional variations in wall displacement amplitude and direction, depending on the blood flow direction and curvature geometry.

### Characterization of Wall Motion Dynamics Using a Fast Fourier Transform Based Spectral Analysis

**Fig 4:**
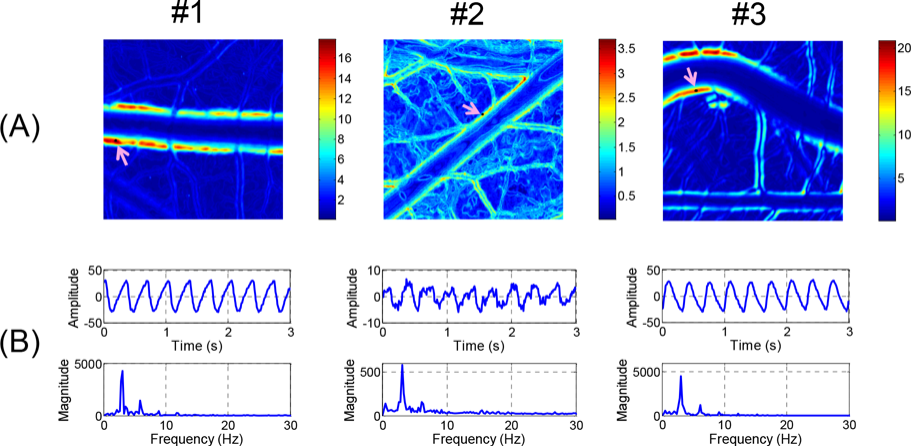
RMS amplitude maps and waveform analysis. (A) Local variations of RMS amplitude corresponding to artery images in Fig 3 are presented in false color maps. (B) Time-amplitude plots and frequency spectra obtained from FFT analysis at selected points in RMS amplitude maps are displayed.

**Fig 5:**
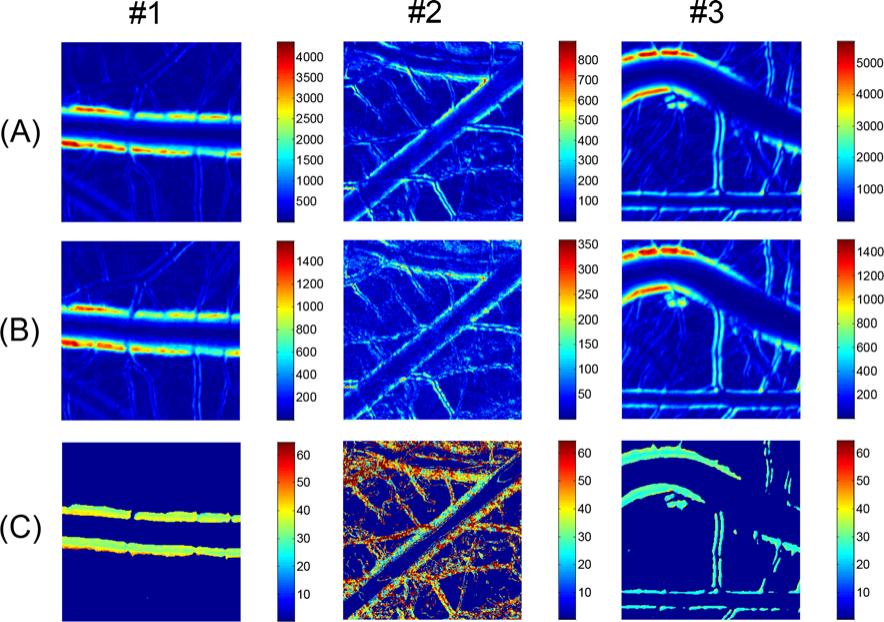
Harmonic amplitude maps of arterial wall motion. Local variations of magnitude of (A) fundamental and (B) second harmonic components corresponding to artery images in Fig 3 are presented in false color maps. (C) Maps for fundamental to second harmonic magnitude ratio obtained from (A) and (B). Color bars in (C) indicate percentage of ratio.

**Fig 6:**
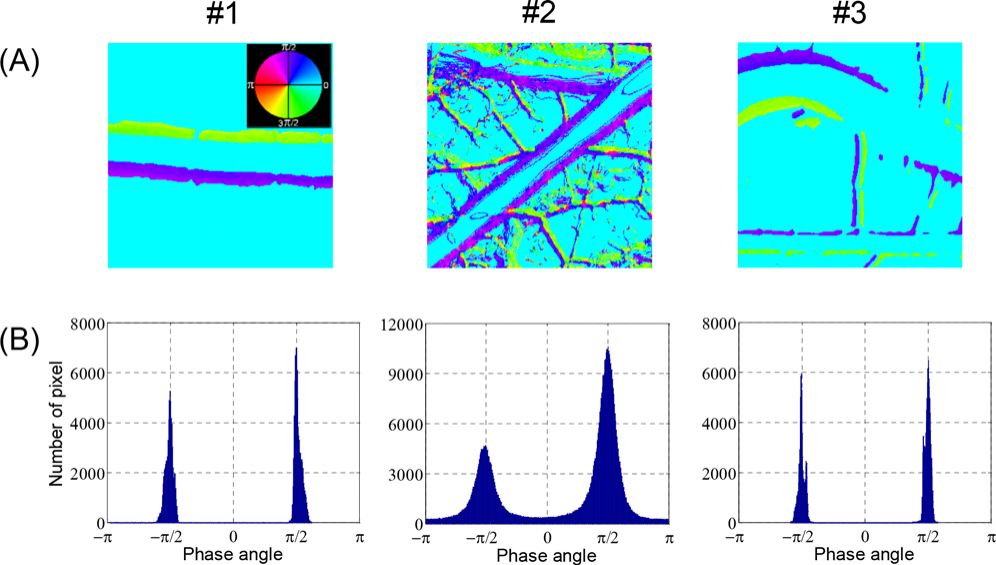
Phase angle maps of arterial wall motion. Local variations in phase angle of fundamental component corresponding to Fig 5A are displayed in (A) continuous phase maps and (B) histograms.

The changes in the brightness of each pixel in the video clips of the artery images in Fig 3 were analyzed by the FFT method. The analysis results are presented as false color image maps in Figs 4 and 5. The maps of the RMS amplitude in Fig 4A reflected the regional variation of wall motion because the RMS amplitude was higher near the vessel boundary with stronger wall motion. The RMS amplitude was not qualitatively correlated with the displacement of the wall movement in physical dimension, but it was possible to estimate relative variation of the wall movement, as shown in Figs 3A and 4A. In the case of the curved artery in which marked regional variations were observed, the RMS amplitude map well reflected the systolic and diastolic variation of arterial boundary. Fig 4B shows the brightness waveforms at the pixels of the vessel edges pointed by arrows in Fig 4A and the corresponding frequency spectra analyzed through the FFT technique. In the brightness waveform with outward wall motion at the systolic phase (cases #1 and #2), the ascending slope was less steep than the descending slope, but the opposite was observed for the inward wall motion at the systolic phase (case #3). These observations were caused by the combined effect of more rapid wall movement at systole and darker color in blood vessels than the surrounding medium. The obtained frequency spectra showed the fundamental and second or higher harmonic components. The fundamental frequency corresponded to the heart beat rate of the chick embryos. The appearance and magnitude of the higher harmonics varied among the measurement locations and embryo samples. The magnitude maps of the fundamental and second harmonic components are shown in Fig 5A and 5B, respectively, and their ratios in the regions above 6 dB for the maximum RMS amplitude are shown in Fig 5C. The maps of the fundamental to second harmonic ratios showed limited regional and individual differences in wall motion harmonics. The phase angle maps of the fundamental component in Fig 6A well presented directional information on wall movement. The angular difference (purple and yellowish green) observed in case #1 indicated that the translational motion in the lateral direction is dominant in this artery, but the same angle (purple color) in both sides of the same artery (case #2) indicated that arterial expansion and contraction are the dominant wall motions in this region. The curved region shown in case #3 had a complex wall motion accompanied by a cyclic change in radius of arterial curvature. The histograms in Fig 6B show that the phase angle maps had two main groups that were almost 180° out of phase, thereby suggesting that wall motion in the arteries had two main directivities, which were in opposite directions, during a pulsatile cycle.

### Arterial Bifurcation

**Fig 7:**
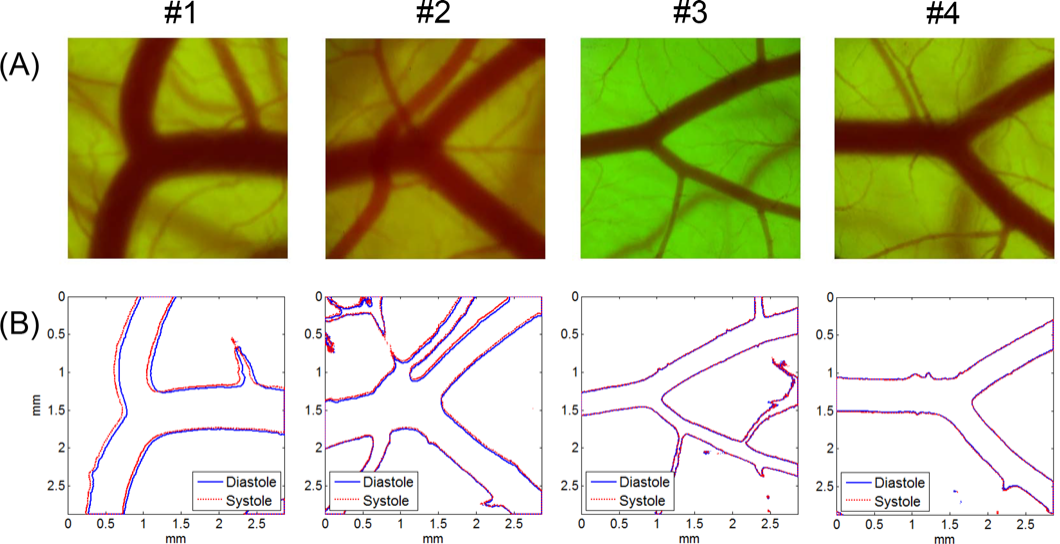
Microscopic CAM artery images and vessel boundaries at systole and diastole (bifurcated arteries). (A) Arterial bifurcation images in CAMs and (B) corresponding vessel boundaries detected at peak systole and late diastole. Artery images were obtained from four *ex ovo* samples (#1: HH 36; #2: HH 37; #3; HH 36; #4: HH 36). Microscope magnification was ×40. Image size was 1,200 pixels × 1,200 pixels.

**Fig 8:**
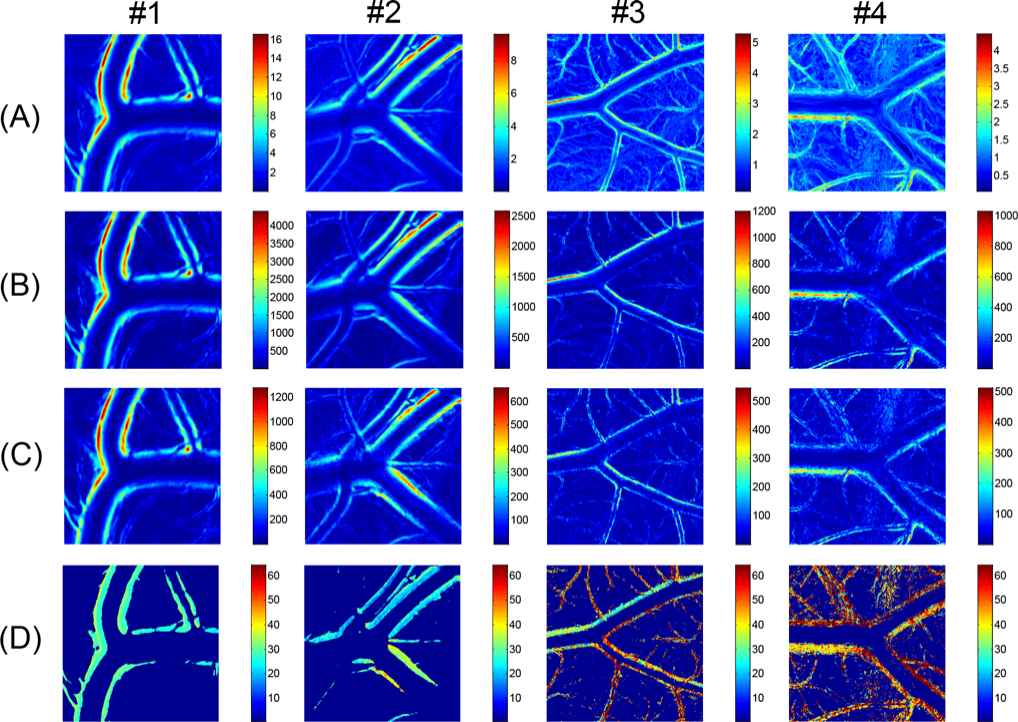
RMS and harmonic amplitude maps of arterial wall motion (bifurcated arteries). FFT analysis results of arterial wall dynamics corresponding to arterial bifurcation images in Fig 7. (A) Local variations in RMS amplitude, and magnitude of (B) fundamental and (C) second harmonic components are displayed in false color maps. (D) Maps for fundamental to second harmonic magnitude ratio obtained from (B) and (C). Color bars in (D) indicate percentage of ratio.

**Fig 9:**
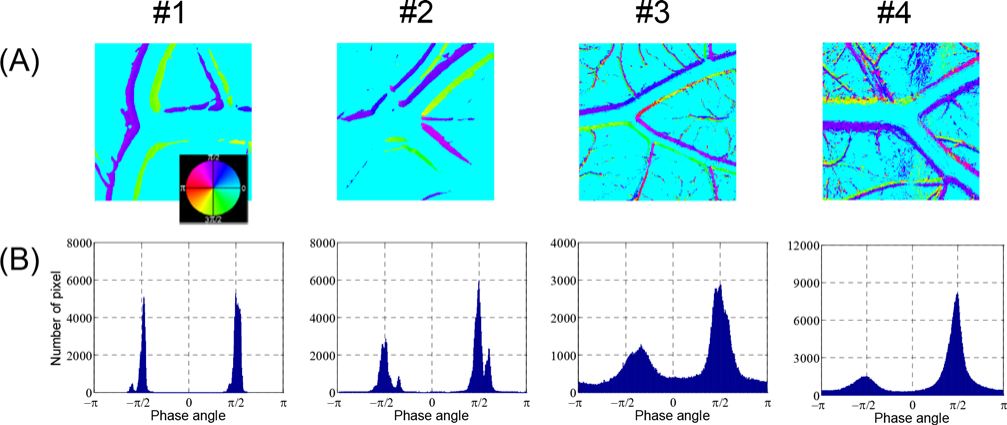
Phase angle maps of arterial wall motion (bifurcated arteries). Local variations in phase angle of fundamental component corresponding to Fig 8A are displayed in (A) continuous phase maps and (B) histograms.

The video images of the arterial bifurcation obtained from four different *ex ovo* samples were analyzed using the techniques for vessel boundary detection and the FFT-based spectral analysis (Figs 7 and 8, respectively). The blood flow direction was from left to right (from one mother artery into two daughter vessels), but the flow was in the opposite direction in case #1. All arterial bifurcations presented in Fig 7 were similar in shape, except for the artery in case #1, which had a larger bifurcation angle. The artery size was larger and the wall motion was more active in cases #1 and #2 than in cases #3 and #4. The RMS amplitude maps in Fig 8A well represented the local variations of the vessel wall movement in the vicinity of the arterial bifurcation. According to the results, the distribution pattern of the wall motion amplitude near the bifurcation varied among the arteries and between the daughter arteries. The overall distribution patterns observed in the RMS amplitude maps appeared similar to the harmonic components in Fig 8B and 8C. However, Fig 8D shows considerable local variations in the magnitude ratio between the fundamental and second harmonic components in the most of the cases except for case #1. The phase angle maps of the fundamental component in Fig 9A show complex local variations in the phase angle of the cyclic wall motion near the arterial bifurcations due to the shifted phase angles distributed near the two main phase angles that were 180° out of phase, as shown in the histograms in Fig 9B. Especially, the histogram for the case #2 artery well represents the phase shift phenomenon because it contained less noise components from the surrounding small vessels compared with cases 3# and #4.

### Wavelet Transform

**Fig 10:**
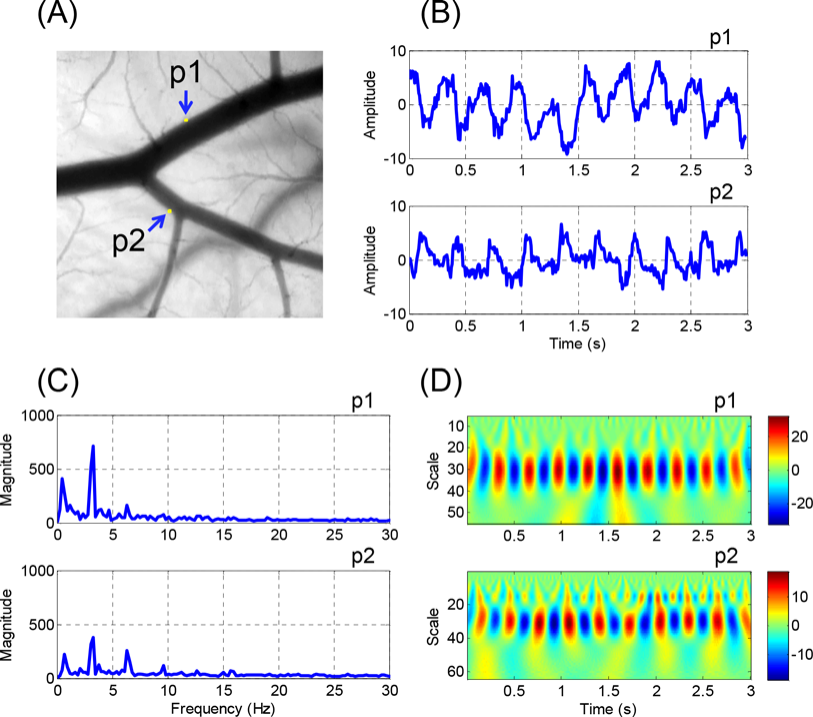
Representative results of wavelet transform analysis. (A) Arterial bifurcation of case #3 is presented as a gray scale image. Pixel brightness waveforms and FFT frequency spectra at points of interests, p1 and p2, are presented in (B) and (C), respectively. (D) Wavelet scalograms at corresponding points are shown. Pseudo-frequencies for fundamental and second harmonic frequencies (3.2 and 6.4 Hz) in the FFT spectra correspond to 30.5 and 15.2 in scale in wavelet scalograms, respectively.

The FFT-based spectral analysis method was useful for investigating the spatial variations in the arterial wall motion, it was not able to provide the time domain information on the variation in the frequency components. Given that the arterial wall motion in CAM did not consistantly showed perfect periodicity because of the bulk movement of the extraembryonic media caused by the aperiodic irregular embryonic movement, additional analysis is required for time-frequency representation. Fig 10 shows the results of a sample analysis using CWT, which is a common approach for time-frequency analysis. The points of interest in Fig 10A, p1 and p2, were selected from the outer walls of the two daughter vessels. Fig 10B shows the time-amplitude waveforms at p1 and p2. The CWT analysis results (Fig 10D) showed that the fundamental component was dominant throughout the whole pulsatile cycles for 3 seconds at p1 and p2. In Fig 10D, higher frequency components were not noticeable at p1 during the measurement period. However, at p2, the second harmonic component was observable and became stronger during the latter part of the measurement period. The pseudo-frequency components observed in the CWT analysis results were in good agreement with the frequency spectra analyzed by the FFT technique (Fig 10D).

## Discussion

We aimed to establish an *in vivo* experimental model suitable for investigating pulsatile variation of an arterial geometry. For this purpose, we investigated the feasibility of using chick CAM model as a source of an *in vivo* arterial system with various geometries. The geometric variations and wall motion dynamics of the arteries were imaged by optical microscopy and estimated using boundary detection and spectral analysis techniques. The analysis results showed that high-fidelity information on the spatio-temporal variations in the arterial geometry dynamics could be obtained from various arteries with different sizes and shapes in the chick CAMs. Compared with other experimental methodologies that detect and analyze arterial wall motion [16, 40–42], our method is relatively simple in terms of image acquisition and data processing and is suitable for estimating local variations in the frequency components of the wall motion dynamics. The CAM artery model can provide useful information for studying fluid-structure interaction in cardiovascular system and hemodynamic etiology of arterial plaque formation, and also serve as a tool for evaluating the effect of arterial wall motion modulators.

By performing the boundary detection process, the 2D geometries of the arteries were precisely measured at the micron scale, as shown in Figs 2, 3 and 7. This process can serve as a useful tool for investigating geometric changes of an artery at different instances during a pulsatile cycle. The vessel boundaries detected at the peak systole and late diastole were considered to reflect the maximum wall displacements during a pulsatile cycle, and may provide important information for studies on the hemodynamic forces acting on arterial wall, such as WSS. However, the phase shift phenomenon of the fundamental component observed in the vicinity of the bifurcations (Fig 9A) implies that the direct comparison of the instantaneous geometries alone is insufficient for the interpretation of arterial wall dynamics. Therefore, a spectral analysis of local wall movement is critically required to obtain highly precise information on wall motion dynamics.

The arterial wall motion is subjected to three directional components, namely, radial, longitudinal, and circumferential directions [43]. However, the *in vivo* measurement of arterial wall displacement in all three directions is difficult with currently available imaging technology. In most studies, the apparent wall displacement in the radial direction is commonly used as a wall motion parameter because of its ease of measurement, whereas the measurement of the longitudinal and circumferential movements is still in the research stages [44–45]. In the present study, we performed spectral analysis of the pixel brightness variation in optical images caused by the combined motions of all directional movements. Our analysis results can provide qualitative and comparative information, but not quantitative values in physical dimension. Nevertheless, the FFT and wavelet analysis techniques help us obtain useful information for interpreting wall motion dynamics, such as the information on spatio-temporal variations of the harmonic components and phase angle.

The frequency harmonics of the WSS waveform have critical functions in the regional development of vascular inflammation and atherosclerosis [46]. Given that the WSS waveform is closely related to the wall motion waveform [47], the spectral analysis method for the arterial wall dynamics can be a potential tool for basic studies on the estimation of WSS frequency harmonics. The local variations in phase angle of the fundamental component observed near arterial bifurcations (Fig 9A) might be caused by the differences in the local hemodynamics and geometries. However, the underlying mechanisms require clarification. The complex regional variations in the harmonic components and phase angles in the vicinity of the arterial bifurcation may be related to the formation and development of arterial diseases, such as stenosis and aneurysm. Therefore, further systematic studies on the relationship between arterial geometry and the local variations of the frequency harmonics and phase angle are required.

The present study was mainly focused on investigating the feasibility of the *ex ovo* chick CAM artery model for studying basic principles in arterial wall motion. The results suggest that this model enables easier observation and investigation of arterial wall dynamics via optical microscopic imaging similar to that observed in human arteries via ultrasound and magnetic resonance imaging. However, there are some limitations in our current experimental setup and instrumentation. Because embryo culture and image acquisition in our experimental setup were carried out at independent instrumental spaces with different humidity and temperature conditions, these environmental stresses might cause unexpected changes in cardiovascular physiology of the embryo samples and measurement artifacts. Therefore, it is desirable to use a microscope incubation chamber that allows whole thermalization of the microscope system and embryo samples. Arteries are considered to have bulk motion in longitudinal direction but no in lateral direction. The waves of cardiac muscle movement in a chick embryo, however, may propagate in extraembryonic media to produce surface waves on the CAM, which may cause weak up-and-down movement of the CAM arteries in lateral direction. Employing three-dimensional imaging techniques with high spatiotemporal resolution such as high-frame rate optical coherence tomography is desirable to avoid the measurement artifacts in two-dimensional imaging that may arise from the lateral movement. High-frequency ultrasound imaging may also be useful for this purpose under the conditions that ultrasound radiation energy and the acoustic coupling media do not affect embryo’s physiology. Also, optical coherence tomography and ultrasound imaging enable simultaneous measurements of arterial geometry changes and blood flow velocity by employing Doppler velocimetry, which is useful for studying WSS variation. As validation and application studies, evaluation of arterial wall motion responses to pharmacological modulators, physiological changes and physical stimuli based on statistical analysis is necessary. It is hoped that our feasibility case study will stimulate other investigators in the fields such as cardiovascular imaging, pharmacology and embryology to become involved in further systematic studies of the CAM artery model.
